# Measuring Heart Rate Accurately in Patients With Parkinson Disease During Intense Exercise: Usability Study of Fitbit Charge 4

**DOI:** 10.2196/51515

**Published:** 2023-12-08

**Authors:** Giulia Colonna, Jocelyn Hoye, Bart de Laat, Gelsina Stanley, Alaaddin Ibrahimy, Sule Tinaz, Evan D Morris

**Affiliations:** 1 Department of Radiology and Biomedical Imaging Yale University New Haven, CT United States; 2 Department of Psychiatry Yale University New Haven, CT United States; 3 Department of Neurology Yale University New Haven, CT United States; 4 Department of Biomedical Engineering Yale University New Haven, CT United States

**Keywords:** Fitbit, heart rate measurements, Parkinson disease, exercise, accuracy, intensity, heart rate, wearable, neurodegenerative disease, aerobic exercise, physical exercise, program, device

## Abstract

**Background:**

Parkinson disease (PD) is the second most common neurodegenerative disease, affecting approximately 1% of the world’s population. Increasing evidence suggests that aerobic physical exercise can be beneficial in mitigating both motor and nonmotor symptoms of the disease. In a recent pilot study of the role of exercise on PD, we sought to confirm exercise intensity by monitoring heart rate (HR). For this purpose, we asked participants to wear a chest strap HR monitor (Polar Electro Oy) and the Fitbit Charge 4 (Fitbit Inc) wrist-worn HR monitor as a potential proxy due to its convenience. Polar H10 has been shown to provide highly accurate R-R interval measurements. Therefore, we treated it as the gold standard in this study. It has been shown that Fitbit Charge 4 has comparable accuracy to Polar H10 in healthy participants. It has yet to be determined if the Fitbit is as accurate as Polar H10 in patients with PD during rest and exercise.

**Objective:**

This study aimed to compare Fitbit Charge 4 to Polar H10 for monitoring HR in patients with PD at rest and during an intensive exercise program.

**Methods:**

A total of 596 exercise sessions from 11 (6 male and 5 female) participants were collected simultaneously with both devices. Patients with early-stage PD (Hoehn and Yahr ≤2) were enrolled in a 6-month exercise program designed for patients with PD. They participated in 3 one-hour exercise sessions per week. They wore both Fitbit and Polar H10 during each session. Sessions included rest, warm-up, intense exercise, and cool-down periods. We calculated the bias in the HR of the Fitbit Charge 4 at rest (5 min) and during intense exercise (20 min) by comparing the mean HR during each of the periods to the respective means measured by Polar H10 (HRFitbit – HRPolar). We also measured the sensitivity and specificity of Fitbit Charge 4 to detect average HRs that exceed the threshold for intensive exercise, defined as 70% of an individual’s theoretical maximum HR. Different types of correlations between the 2 devices were investigated.

**Results:**

The mean bias was 1.68 beats per minute (bpm) at rest and 6.29 bpm during high-intensity exercise, with an overestimation by Fitbit Charge 4 in both conditions. The mean bias of the Fitbit across both rest and intensive exercise periods was 3.98 bpm. The device’s sensitivity in identifying high-intensity exercise sessions was 97.14%. The correlation between the 2 devices was nonlinear, suggesting Fitbit’s tendency to saturate at high values of HR.

**Conclusions:**

The performance of Fitbit Charge 4 is comparable to Polar H10 for assessing exercise intensity in a cohort of patients with PD (mean bias 3.98 bpm). The device could be considered a reasonable surrogate for more cumbersome chest-worn devices in future studies of clinical cohorts.

## Introduction

### Background

Parkinson disease (PD) is the second most common neurodegenerative disease and affects approximately 1% of the world’s population [1]. The main symptoms characterizing this disease are bradykinesia, rigidity, tremor, and postural instability as well as nonmotor symptoms, such as anxiety, depression, sleep disturbance, and fatigue. Evidence suggests that aerobic physical exercise can be beneficial in mitigating motor symptoms and slowing the progression of the disease [1-3].

The extent of benefits observed differs depending on the exercise type, intensity, and duration. Various recent clinical trials have concluded that moderate- to high-intensity exercise several times per week, when maintained over extended periods, is associated with slower deterioration of motor symptoms in PD [4,5].

Since different types of exercise interventions at varying intensity levels are used in clinical trials for PD and other clinical populations, there is a need for objective methods to monitor the intensity of physical activity. The popularity of wearable devices has grown, as they have become more affordable, useful, and less intrusive [6]. However, it is still necessary to establish the reliability of these devices in tracking physiological parameters during both clinical trials and personal use.

Wearables can measure many different parameters, such as heart rate (HR), number of steps, calories expended, and quality of sleep. HR is considered an essential indicator of physiological adjustment and intensity of effort [7]. HR is correlated linearly with moderate- and vigorous-intensity physical exercise and is a valuable option to monitor the intensity of activities (eg, cycling, swimming, and activities that are not ambulatory) that may not be easily measured with other methods, such as accelerometry [8]. Following the American Heart Association guidelines, vigorous exercise intensity can be defined as 70% to 85% of the maximum HR [9]. Many different tools can be used to assess HR, such as electrocardiogram (ECG) monitors; chest, shoulder and arm straps; and wrist watches.

In this study, we compared Polar H10 (Polar Electro Oy) and Fitbit Charge 4 (Fitbit Inc), two types of wearable devices that are commonly used as activity trackers and HR monitors.

The Polar Heart rate (referred to as Polar H10 in this paper) is a chest strap that uses ECG technology to measure the R-R interval. The Polar H10 has been reported to be highly correlated to 3-lead ECG Holter monitor (*r*=0.997) and is now considered the gold standard for assessment of R-R intervals in sports settings [10] as well as maintaining a certain accuracy in older adults affected by cardiac disease [11]. Despite its accuracy, the Polar H10 monitor is often perceived as too cumbersome to use, and it may cause discomfort, especially for older people [12]. Since it needs to be strapped across the sternum, it may be difficult to tolerate over extended periods [12]. On the other hand, as a wrist-worn tracker, Fitbit Charge 4 (referred to as Fitbit in this paper) is more convenient and comfortable to wear, and it promotes patient compliance in studies requiring prolonged measurements [13]. According to Düking et al [14], wrist-worn wearables, being able to provide direct biofeedback, have the potential to increase participation in exercise.

Fitbit Charge 4 is a recent model of the Fitbit Charge HR series, released in March 2020. It is a wrist-worn device that detects HR by measuring the volume changes in blood vessels via a photoplethysmography (PPG) optical HR sensor [7]*.* Originally designed to motivate people to exercise, Fitbits are increasingly used as measurement devices in physical activity and health promotion research; they are also used for guiding patient-health professional interactions [15]*.*

Fitbits are commonly used for research purposes [16], but there is no consensus in the scientific literature regarding their accuracy for quantifying HR and confirming high intensity. Some authors have concluded that the device provides values of HR comparable with criterion field-based measures, while others have found that Fitbit does not satisfy the validation criteria, especially during higher exercise intensities [17]. There is even less information on the accuracy of the device in older individuals affected by chronic diseases [13]. Further evaluation is needed.

Ensuring the accuracy of exercise session intensity assessment is crucial in clinical studies involving sports activities and clinical populations. It has yet to be determined if Fitbit has comparable accuracy to Polar H10 in selecting high-intensity sessions in patients with PD or in clinical populations, generally.

### Objective

This paper aims to compare Fitbit Charge 4 to Polar H10 for monitoring HR, confirming high-intensity exercises in patients with PD engaging in an intense exercise program and supporting its potential utility as an activity tracker for use in large clinical trials with similar cohorts.

## Methods

### Population and Study Design

The data for this paper were acquired as part of a larger study to evaluate the role of physical exercise in PD, in which we sought to confirm exercise intensity by monitoring HR. In brief, a total of 11 participants, 6 of whom were male, aged 58-68 years, all with early-stage PD (defined according to the Movement Disorder Society criteria [18]) were recruited. Participants were excluded based on the criteria of the larger study, as follows: (1) heavy drinking or illicit drug use, (2) neurologic or psychiatric disorders other than PD, (3) diseases interfering with one’s ability to exercise, (4) contraindication to positron emission tomography or magnetic resonance imaging scans, (5) severe motor symptoms (tremor and dyskinesia) likely to introduce motion artifacts in imaging data, (6) unsafe to come off dopaminergic medication, (7) BMI>30 (practical issues with the neuroimaging equipment), (8) extreme exercisers, and (9) Hoehn and Yahr disease stage>2 (stage 2 corresponds to mild bilateral disease with intact balance [19]). None of the participants had a history of arrhythmias or any other cardiac conditions that could potentially affect the measurements of the devices. Additionally, none of them were under medication, such as AV nodal blockade therapy, which might have altered the HR detection capabilities of Polar H10 and Fitbit Charge 4.

Each participant engaged in exercise for a period of 6 months, with at least 3 Beat Parkinson’s Today (BPT) exercise sessions per week. The BPT program is an established exercise program that combines those aspects that have been shown to be the most effective in achieving symptom improvement in PD [20], such as high-intensity interval training and boxing [21,22]. Each session included a mix of these 2 activities, which could be adapted to any fitness level. Functional interval training circuits were designed specifically to improve explosiveness, gait, and strength. Trainers continuously encouraged participants to work at their own personal level of maximum intensity while attempting to reach a target HR.

To compare the performances of devices, participants were equipped with a Fitbit and a Polar H10. The Fitbit was worn on the wrist and positioned a finger’s width above the wrist bone, as recommended by the company. The wrist-worn tracker was situated on the side less affected by PD. The Polar HR sensor was placed over the sternum and held in place by a chest strap. The exercise sessions lasted 60 minutes, including warm-up, at least 20 minutes of high-intensity exercise, boxing, and cool-down. When unable to attend classes, participants were encouraged to exercise on their own and monitor their HR using both wearables.

### Ethical Considerations

All procedures with human subjects were approved by the Human Investigations Committee of Yale University (approval number 2000028563).

### HR Data Processing From Wearables

The data from Fitbit Charge 4 were collected by synchronizing each watch with an anonymized web-based account for each participant and downloaded via the mobile app Pulse Watch [23]. The data from Polar H10 were exported using the mobile app Elite HRV. The Fitbit data were sampled every minute by the Pulse Watch app.

The data from Elite HRV were converted from R-R intervals to beats per minute (bpm) and were filtered using a Python function called Butterworth filter to remove high-frequency artifacts. The order was set to 5, and the filter was applied at a frequency of 0.1 Hz. The resulting data set was in units of bpm collected per 10 seconds and was then sampled every minute. The Fitbit data were sampled every minute by the Pulse Watch app.

The validity of Fitbit was compared with Polar H10 in terms of averages between single data points. For each session, HR averages (HRμ) from both the first 5 minutes, generally coinciding with the rest period before the start of the exercises, and from the 20 minutes of the highest-intensity exercise were calculated. The 20 minutes of the highest-intensity exercise were extracted from the data by calculating the HR average for consecutive 20-minute intervals, starting from the initial interval, then shifting forward by 1 minute at a time, and then picking the highest average.

The session averages were then collected, and the values from the 2 different devices were paired. Data alignment, filtering, and calculations were performed with Python (Jupyter Notebook). The precise timestamps for recordings from both devices were available and were used for data alignment. Out of a possible 792 paired sessions, a total of 596 paired sessions were obtained. Data were lost due to multiple factors—nonattendance of the participants, misplacement of the devices, and injuries. The data obtained were contributed roughly equally by all the participants, with a mean of 54 sessions per participant (Table 1)*.*

To ensure the capability of Fitbit, compared to Polar H10, in evaluating the intensity of an exercise session, every HR average (HRμ) was normalized (HR_N_) by different percentages of each participant’s own theoretical maximum HR (HR*_th/max_*) using the following formula:



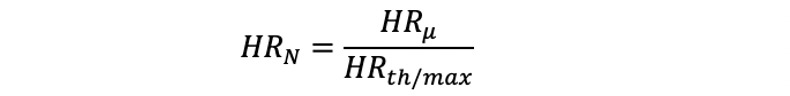



where the term HR*_th/max_* is given by the following: HR*_th/max_* = 220 – age

A session was considered positive if the ratio was >1 and negative if the ratio was <1. Measurements with the Polar H10 were considered to be the gold standard. Therefore, a session was a “true positive” if the ratio was >1 for both devices, “true negative” if the ratio was <1 for both devices, “false positive” if the ratio was >1 for Fitbit and <1 for Polar H10, and “false negative” if the ratio was <1 for Fitbit and >1 for Polar H10.

**Table 1:** Mean difference and limits of agreement (LoA) calculated individually by participant.

Participant	Age (years)	Recorded sessions, n	Mean difference at baseline conditions (bpm^a^)	LoA (bpm)	Mean difference at high-intensity exercise conditions (bpm)	LoA (bpm)
Participant 1	58	81	2.77	–20.4 to 14.8	–2.52	–17.3 to 18.7
Participant 2	73	43	–1.25	–19.4 to 16.9	–11.26	–13.9 to 35.7
Participant 3	60	55	1.93	–26.4 to 30.1	0.42	–31.6 to 30.8
Participant 4	63	38	–1.35	–16.2 to 13.5	–5.93	–4.65 to 16.5
Participant 5	63	56	0.05	–12 to 11.9	–16.37	–7.46 to 37.6
Participant 6	76	98	0.61	–14.1 to 15.3	–15.94	–9.50 to 41.3
Participant 7	56.3	61	–6.94	–31.3 to 17.4	9.44	–36.2 to 17.3
Participant 8	68	28	–8.01	–20.4 to 4.40	–12.14	–10.2 to 25.2
Participant 9	66.6	43	–1.25	–25.4 to 22.9	–5.65	–6.18 to 17.5
Participant 10	66.6	56	–2.71	–21.9 to 16.1	–20.10	–12.9 to 49.3
Participant 11	68	36	0.82	–18.3 to 19.9	5.91	–27.4 to 15.5

^a^Bpm: beats per minute.

### HR Data Comparisons Between Wearables

All statistical analyses were conducted using Microsoft Excel 16 and MatLab (Mathworks, 2018b). To guarantee consistency of the results and the calculations of HR averages at baseline and high-intensity conditions, exercise sessions lasting less than 20 minutes were excluded from the analysis.

A Bland-Altman plot was used to evaluate the agreement between the two methods of measurement, with the limits of agreement (LoA) defined as the mean difference plus or minus 1.96 SD of the difference. The mean difference in HR between the Fitbit and Polar H10 was calculated for the cohort and every participant, both at baseline (rest period) and during high-intensity conditions. A final average of the two mean differences was assessed and considered to be the mean bias. Evaluations were conducted for both intrasubject and intersubject variability (Table 1).

The relationship between both devices at baseline and during high-intensity conditions was determined. The quality of the linear fit was assessed with the *R*^2^ value, considering the data from baseline and high-intensity conditions separately. Subsequently, the entire data set of session HR averages was fitted with linear, logarithmic, negative exponential, and sigmoid model functions to explore different types of relationships between the two devices. The Akaike information criterion (AIC) value was used to assess the relative quality of the fits.

### Sensitivity and Specificity

To determine Fitbit’s sensitivity, specificity, positive predictive value (PPV) and negative predictive value (NPV) in identifying high-intensity exercise sessions, European and American Guidelines cutoffs were applied [24]. Polar H10 was considered to be the gold standard.

To illustrate the sensitivity and specificity of the Fitbit, a receiver operating characteristic (ROC) curve was created for different levels of target HR. The area under the curve (AUC) was used as an indicator of Fitbit’s capability for distinguishing between high-intensity and low-intensity exercise sessions.

## Results

**Figure 1:**
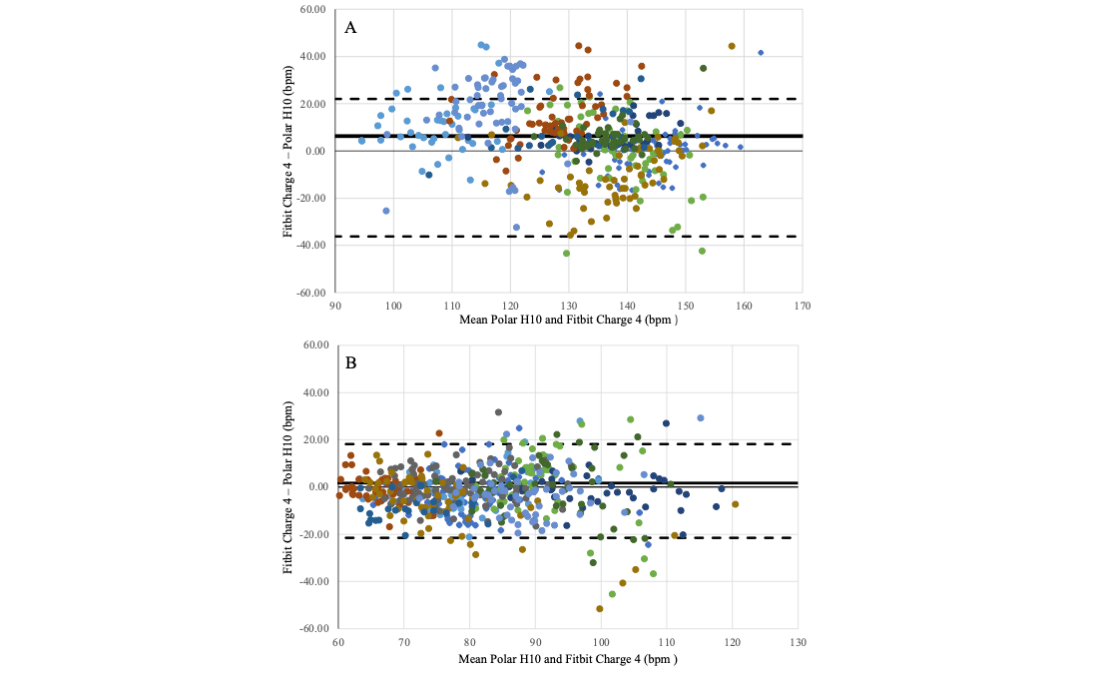
Bland-Altman plots for the difference in heart rate average by every session. The solid line represents the mean bias. The dashed lines represent the limits of agreement. Dots of different colors represent different participants. (A) Bland-Altman plots at baseline conditions and (B) at high-intensity conditions. Bpm: Beats per minute.

The Bland-Altman plots revealed that the mean bias between the Fitbit and Polar H10 was 1.68 bpm (LoA –21.52 bpm to 18.8 bpm) at baseline conditions and 6.29 bpm (LoA –22.02 bpm to 36.2 bpm) under high-intensity exercise (Figure 1). Overall, the mean bias of the Fitbit was 3.98 bpm.

**Figure 2:**
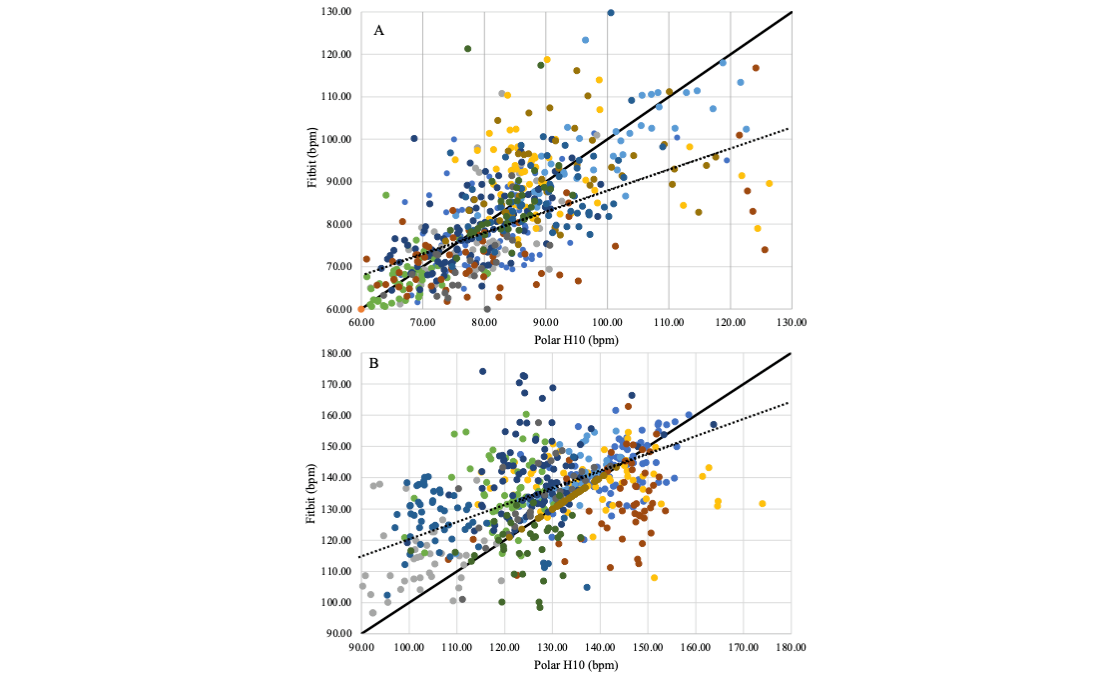
Linear correlations between heart rate measurements of Fitbit Charge 4 and Polar H10. The dots represent each exercise session, the solid line represents the ideal correlation (X=Y), and the dashed line is the observed correlation. Dots of different colors represent different participants. (A) Linear correlation plot at baseline and (B) during high-intensity exercise. Bpm: Beats per minute.

When data from baseline and high-intensity conditions were taken separately, the linear correlations were, respectively, as follows: *R*^2^=0.45 (baseline); *R*^2^=0.23 (high-intensity condition; Figure 2).

**Figure 3:**
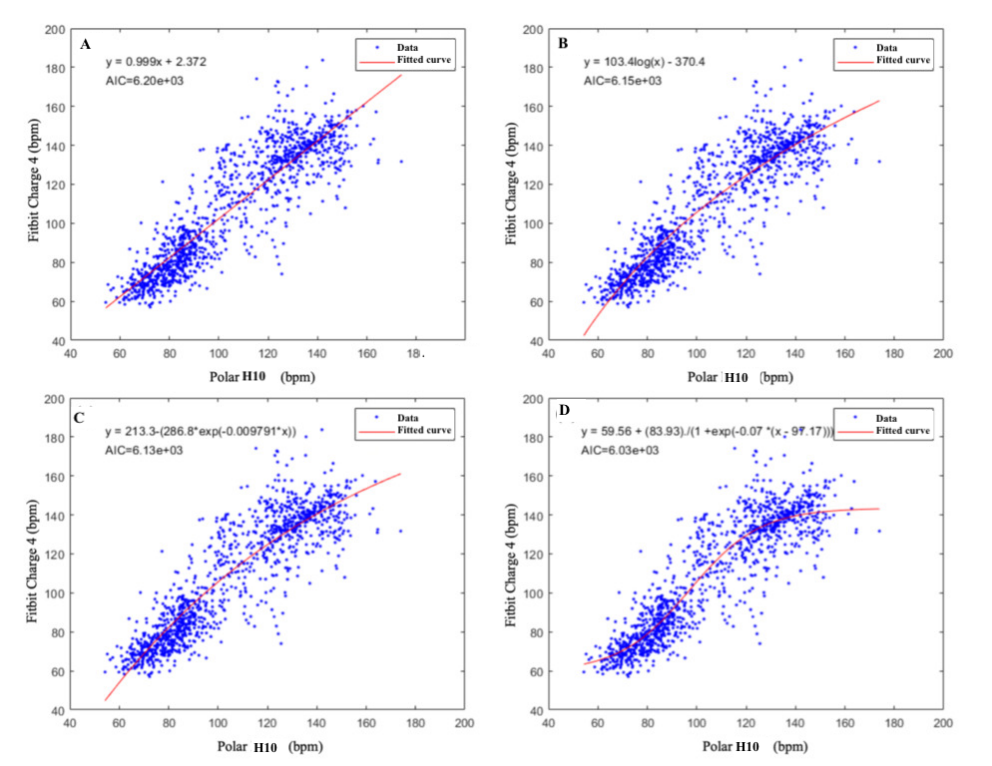
Model fits of Fitbit Charge 4 versus Polar H10. (A) linear, (B) logarithmic, (C) A-exponential, and (D) sigmoid. The blue dots represent heart rate averages from every session; the red solid lines represent the fitted curves. Akaike information criterion (AIC) values and fitting equations are shown as well. Bpm: Beats per minute.

The fit of the combined high-intensity and baseline data to a sigmoid model resulted in the lowest AIC value (AIC=6.03e+03; Figure 3).

When the mean differences were calculated individually by participant, there was evidence of intersubject and intrasubject variability (Table 1).

With 70% of maximum HR as the tailored threshold indicating high intensity and considering Polar H10 as the gold standard, the sensitivity, specificity, PPV, and NPV of the Fitbit were 97%, 11%, 89%, and 35%, respectively. With 85% of the maximum HR as the threshold, the sensitivity, specificity, PPV, and NPV of the Fitbit were 78%, 56%, 62%, and 73%, respectively (Table 2).

**Figure 4:**
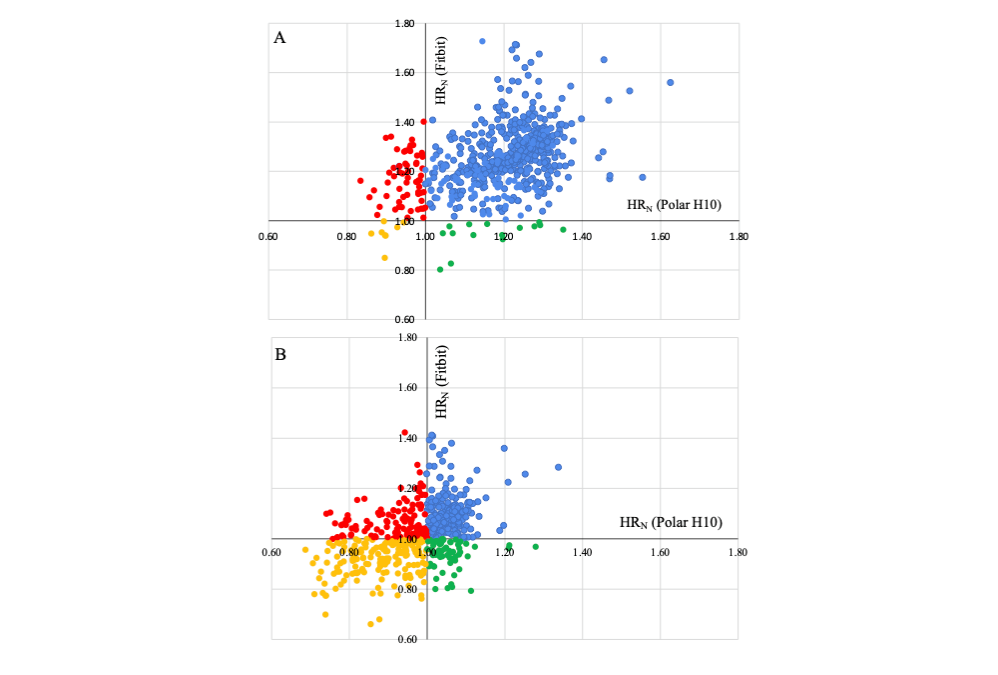
(A) The number of true positives, false positives, true negatives, and false negatives based on a target of 70% of maximum heart rate (HR) and (B) based on a target of 85% of the maximum HR. The y-axis and x-axis are normalized; the dots represent the normalized value of each exercise session. The blue dots represent the true positives (>1 for both devices); the red dots represent the false positives (>1 for Fitbit Charge 4 and <1 for Polar H10); the yellow dots represent the true negatives (>1 for both devices); and the green dots represent the false negatives (<1 for Fitbit Charge 4 and >1 for Polar H10).

These indicators of performance at the 2 different thresholds can be visualized graphically in Figure 4. As expected, when the threshold was set higher, the number of true positives decreased, and the sensitivity of the Fitbit decreased.

**Figure 5:**
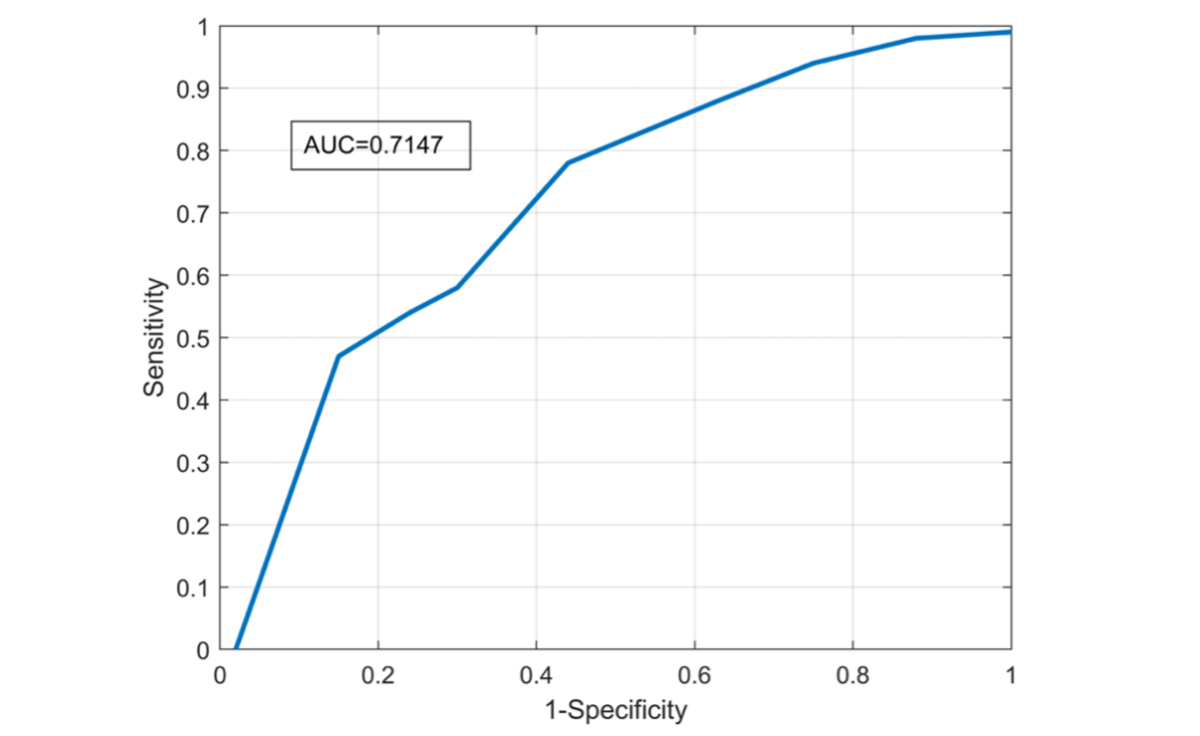
Receiver operating characteristic curve of Fitbit Charge 4 taking Polar H10 as the gold standard. AUC: area under the curve.

The ROC curve (Figure 5) depicts the performance of Fitbit Charge 4 for varying HR thresholds. The AUC was 0.71.

**Table 2:** Sensitivity and specificity of Fitbit Charge 4 given 70% and 85% of the maximum heart rate (HR) as thresholds.

Threshold	True positives, n	False positives, n	True negatives, n	False negatives, n	Sensitivity (%)	Specificity (%)	PPV^a^ (%)	NPV^b^ (%)
70% of the maximum HR	509	63	8	15	97.1	11	89	35
85% of the maximum HR	223	134	172	64	78	56	62	73

^a^PPV: positive predictive value.

^b^NPV: negative predictive value.

## Discussion

### Principal Results

To our knowledge, this is the first evaluation of Fitbit Charge 4 with a population of individuals with PD. We found the bias to be approximately 3.98 bpm during intense exercise. The magnitude of bias in the Fitbit is consistent with a report from a meta-analysis published in 2022 [16] (mean bias 3.39 bpm; LoA –24.3 bpm to 17.53 bpm). Thus, we conclude that the bias of the device, while remaining statistically significant in comparison to more precise devices, like Polar H10, is not influenced by PD and its associated symptoms, such as tremors and rigidity. Rather, it may be a limitation of the manufacturer’s software process for processing data from the PPG technology. The algorithm adopted by the company to estimate HR from the PPG measurements is confidential, but some authors suggested that it may rely on the P-P intervals of the PQRST wave (atrial contractions). Not all the P peaks are consistently present and detectable when the frequency increases. ECGs, on the other hand, register the full PQRST wave and quantify the final HR by using the interval between R peaks, making it less prone to these artifacts. R peaks (ventricular contractions) are the best detectable peaks in the PQRS wave. Consequently, the calculation of the HR by PPG can be influenced by inaccurate sampling and recording of the P peaks [7]. Moreover, the performance of Fitbit, as with all wrist-based devices, is dependent on correct placement on the wrist. Therefore, when wrist movements are greater, measurement accuracy may be compromised. Devices like Polar H10, which are placed on the chest, may be less prone to movement artifacts [25].

Bland-Altman analysis showed that Fitbit tended to overestimate the values of HR compared to Polar H10 in high-intensity conditions. This result contradicts most of the previous reports [7,15,16]. The overestimation could be due to the peculiar characteristics of this study population (older adults affected by PD). These 2 conditions are known to potentially increase the heterogeneity of Fitbit accuracy results [16]. In previous studies, Fitbit overestimated time spent on moderate to vigorous activity in clinical populations with functional limitations, compared to the criterion devices [26]. Even though in our cohort, the disease appeared not to have an impact on Fitbit’s magnitude of error, it could have altered the sign of the error. There are some additional conditions of our study to consider. The maximum HR values reached by our participants cannot be compared with those reached by a cohort of young, healthy individuals. If HR values had been higher, we might have observed an underestimation by Fitbit. Another important factor to consider is the particular Fitbit model. The only Fitbit Charge 4 validation study was conducted in 2022 [17], which evaluated the device on 23 young participants (average age 24.2 years) without any underlying health conditions.

The linear correlation between the two devices was poor, especially in high-intensity exercise conditions (baseline: *R*^2^=0.45; high-intensity conditions: *R*^2^=0.23; Figure 2). From our statistical analysis, the sigmoid fit, which resulted in the lowest AIC, best described the relationship between Fitbit and Polar H10 (Figure 3). The tendency of Fitbit to saturate at the highest HR values suggests a diminishing ability of Fitbit to resolve high HR values. This finding is in agreement with the existing literature [27]. The reduced precision of Fitbit in measuring high HR values may be attributed to motion artifacts due to physical movement, particularly those involving arm movements, as well as potential misalignment between the skin and the optical sensor [27]. Another hypothesis suggests that wrist-worn devices may not be as sensitive to sudden changes in exercise intensity [25], which occur frequently in high-intensity interval training, as used in our study. Peripheral resistance is lower at the wrist, which reduces pulse pressure changes and alters blood pulse detection. [25]. Although the sigmoid function was the best fit for the data acquired in this cohort, we caution against using the sigmoid model to extrapolate the relationship between the Fitbit and chest strap HRs beyond the range of HR values acquired in this study. In other words, if future studies in patients with PD seek to acquire Fitbit data only (no chest strap data) and want to use this model to predict the chest strap HR, the authors recommend only applying the model to data with HR in the range of 60 bpm to 160 bpm.

We also examined the ability of Fitbit to discriminate HR during high-intensity sessions, via the ROC curve. An AUC of 0.5 generally indicates no discrimination; an AUC of 0.7 to 0.8 indicates acceptable discrimination; an AUC of 0.8 to 0.9 indicates excellent discrimination; and an AUC of more than 0.9 indicates exceptional discrimination [28]. The Fitbit’s measurement can be considered acceptable (AUC 0.71; Figure 5). Consequently, this device is acceptable in identifying correctly high-intensity exercise sessions and could be used with caution in large clinical trials in patients with PD.

### Limitations

Our study is not without limitations. First, the number of sessions is not equally distributed between participants. Thus, some participants may have exerted a greater impact on the total mean difference between the devices than others, as shown in Table 1.

We considered the first 5 minutes of every exercise session as the baseline, during which participants were instructed to sit and breathe. However, there were instances of participants arriving late or forgetting to activate the device at the start of the session, potentially confounding the baseline measurements. Consequently, the values of HR recorded during baseline conditions may have been artifactually high.

When participants were unable to attend classes, they were encouraged to exercise independently while monitoring HR using both devices. However, during these unsupervised sessions, we were unable to ensure the proper fit of both devices, potentially affecting the accuracy of the measurements obtained.

The data processing involved multiple stages of averaging, ranging from a subsecond level to a per-minute level and ultimately to an exercise-session level. Although this averaging approach allowed us to accomplish the study objectives, it may have potentially compromised the precision and reliability of our comparisons.

Due to the inclusion and exclusion criteria of the previous study, only 11 participants, affected by mild PD were taken into account. It is possible that the outcomes would have been different with the inclusion of participants with severe PD, affected by motor symptoms likely to introduce motor artifacts in wearables data. Given the small number of participants and the peculiar characteristics of the cohort, our findings may not be applicable to all patients with PD. For future studies, it may be crucial to involve participants at more advanced stages of the disease to effectively assess Fitbit’s performance under these conditions.

Lastly, the cohort in our study demonstrated significant intrasubject and intersubject variability, attributed to factors such as age, sex, and physical condition. Intrasubject variability is represented by each participant’s LoA and intersubject variability is depicted as each participant’s HR mean difference (Table 1). The wide LoA observed in the mean bias of Fitbit Charge 4 emphasizes some reasons for cautious interpretations of the results.

### Conclusions

The magnitude of bias and the LoA for Fitbit were consistent with those of previous studies, and the performance of Fitbit fell within the range of 4 bpm, compared to Polar H10 for assessing intense exercise in a cohort of patients with PD. A wrist-worn device, Fitbit, offers clear advantages in terms of wearability and practicality. In future studies involving clinical populations, the device could be considered as a reasonable alternative to the more intrusive chest strap technology.

## Abbreviations

AIC: Akaike information criterion
AUC: area under the curve
Bpm: beats per minute
ECG: electrocardiogram
HR: heart rate
LoA: limits of agreement
NPV: negative predictive value
PD: Parkinson disease
PPG: photoplethysmography
PPV: positive predictive value
ROC: receiver operating characteristic

## Data Availability

The data sets generated and analyzed during the study are available from the corresponding author on a reasonable request.
